# Low Frequency Electrical and Magnetic Methods for Non-Destructive Analysis of Fiber Dispersion in Fiber Reinforced Cementitious Composites: An Overview

**DOI:** 10.3390/s130101300

**Published:** 2013-01-21

**Authors:** Marco Faifer, Liberato Ferrara, Roberto Ottoboni, Sergio Toscani

**Affiliations:** 1 Department of Electrical Engineering, Politecnico di Milano, piazza L. da Vinci 32, 20133 Milan, Italy; E-Mails: marco.faifer@polimi.it (M.F.); roberto.ottoboni@polimi.it (R.O.); sergio.toscani@polimi.it (S.T.); 2 Department of Civil and Environmental Engineering, Politecnico di Milano, piazza L. da Vinci 32, 20133 Milan, Italy

**Keywords:** non-destructive monitoring, FRC, FRCCs, magnetic inductance, fiber dispersion, mechanical performance

## Abstract

Non-destructive analysis of fiber dispersion in structural elements made of Fiber Reinforced Concrete (FRC) and Fiber Reinforced Cementitious Composites (FRCCs) plays a significant role in the framework of quality control and performance prediction. In this paper, the research activity of the authors in the aforementioned field all over the last lustrum will be reviewed. A method based on the measurement of the inductance of a probe to be placed on the specimen will be presented and its progressive development will be described. Obtained correlation with actual fiber dispersion, as checked by means of destructive methods, as well as with the mechanical performance of the composite will also be presented, in an attempt to address the significance of the method from an engineering application perspective.

## 1. Introduction

Since the pioneering early studies on structural applications Fiber Reinforced Concrete (FRC) and Fiber Reinforced Cementitious Composites (FRCCs) [1] the dispersion of the fibers within a FRC structural element was recognized as a crucial issue for the effective development of the toughening action provided by the fibers. It was also recognized quite early that the not infrequently hypothesized randomly uniform dispersion of the fibers within a structural element could hardly be achieved, because of the negative effects that fibers themselves have on the workability of fresh concrete [2]. Because of this, casting of elements made with FRCs could hardly be accomplished without external interventions, such as manual compacting and vibration, which could jeopardize the above recalled random uniformity of fiber dispersion. It is worth here remarking that a poor dispersion of fibers in a structure, affected by casting and processing, is not only a mere technological problem: as a matter of fact spots with a reduced fiber dosage or no fibers at all act as flaws and trigger early failures by activating unpredictable mechanisms, that affect the load-bearing capacity and the structural performance as a whole.

The advances in the technology of Self Compacting Concrete (SCC), in the last two decades, have led to the development of admixtures and innovative concepts about granular packing and mix design criteria which have been instrumental to successfully solve the aforementioned issues for workability and fresh state performance of FRCC and have thus made possible the successful design and casting of Fiber Reinforced Self Compacting Concretes (FR-SCCs, see, e.g., [3]). The major advantage of incorporating fibers into a Self-Compacting Concrete matrix is the achievement, thanks to the elimination of compaction and vibration and to the rheological stability of the same matrix, of a randomly uniform dispersion of fibers within structural elements, not affected by their segregation [4–6]. This requisite is, as mentioned above, of paramount importance for a reliable structural performance of elements made with FRCCs. It has also been recently recognized that, through a suitably balanced performance of the fluid mixture, fibers can be effectively aligned along the casting-flow direction [7–12]. By suitably tailoring the casting process to the intended application, the flow direction of the fresh concrete, along which fibers tend to be aligned, can be made to coincide, as closely as possible, with the anticipated stress pattern (*i.e.*, the direction of principal tensile stresses) within the structural element when in service. This would lead to a better structural efficiency of the material.

In this framework, the development of a time- and cost-effective methodology for non-destructive analysis of fiber dispersion and orientation in FRC structural elements has become a crucial need and its value, from an engineering application point of view, is twofold. On one hand in fact these methodologies should be effectively implemented into the procedures for quality control and acceptance for elements made with FRC, in order to assess the dispersion and orientation of the fibers actually achieved because of the employed, in case tailored, casting process. On the other hand, which is even more interesting and challenging in the authors' opinion, these methodologies should become an effective part of the whole design process, which cannot disregard fiber dispersion related issues and their outcome on the “in-structure” mechanical performance of the material. As a matter of fact, once the correlation between fiber dispersion/orientation and material mechanical properties has been established, by means of suitable testing methodologies, a structural analysis could be performed by assigning material properties featuring a “spatial dispersion”, as it can be inferred, through the aforementioned correlation, from the cartography of the same fiber dispersion and orientation, as garnered from a carefully planned ND testing of the element. This would allow a thorough assessment of the structural performance of the element, as affected by spatial scattering of material properties, resulting from the actual dispersion of fibers within the element. This would provide highly valuable information, also with reference to the assessment of the structural performance of existing elements or in the case when the aforementioned quality control/acceptance procedures would have yielded a dubious result.

In this paper the authors will present a review of different methods they have developed over the past lustrum for non-destructive analysis of fiber dispersion in FRC elements, based on either electrical or magnetic properties of fiber reinforced cementitious composites. A detailed state of the art on the topic will be prefaced, providing a proper framework for the authors' work.

## 2. Non-Destructive Analysis of Fiber Dispersion in FRCCs: Literature Survey

The early works on non-destructive analysis of fiber dispersion in FRC structural elements employed X-ray imaging [13]. Besides the safety concerns related to the use of X-ray equipment on industrial scale, it has to be remarked that such methods, which have been also widely used thereafter by several researchers, mainly as a pre-check of destructive analyses [4,14] are able to provide an immediate visualization of the discrete fiber reinforcement network inside the analyzed region of the element. As a matter of fact, the thickness of the element, in order to have meaningful information from the images, has to be necessarily quite small, as a function of the power of the employed equipment and of the absorption capacity of the material. Furthermore, the unavoidable “loss” of the third dimension may introduce some artifacts in the results of image processing, affecting any quantitative information, e.g. about local fiber density, which could be garnered from it. As a matter of fact, the higher the fiber dosage and the aspect ratio of the fiber, the more influence have these artifacts.

Electrical methods, based on the effects of the fibers on the resistivity/conductivity of the composite material, have received lots of attention in the very last years. Ozyurt *et al.* [15] employed the Alternating Current Impedance Spectroscopy (AC-IS) technique for the detection of fiber dispersion related issues and demonstrated its reliability as well as its sensitivity to fiber orientation, clumping, segregation *etc.* by means of extensive comparison with results obtained from destructive methods. Attempts were also made to address the application of the aforementioned method to industrial scale problems [16]. The method consists of applying to the specimen a voltage excitation over a wide range of frequencies (e.g., 10 MHz–1 Hz) and measuring the amplitude and phase of the flowing current. When considering direct current (DC) or low frequency alternating current (AC), the behavior is practically insulating. However, the impedance Z significantly drops under high frequency alternating current excitation as a result of the displacement current. When the real and imaginary parts of the calculated impedance are plotted on a Nyquist diagram, FRCCs exhibit the so called dual-arc behavior, with two cusps. Each of them represents a local minimum of the imaginary part of the impedance expressed as a function of the real part. The rightmost cusp corresponds to the DC resistance across the electrodes, namely the real part *R_m_* of the impedance, is essentially due to the conductivity *σ_m_* of the matrix. Previous works have shown that it is weakly affected by the presence of the fibers. The leftmost, high frequency cusp is strictly related to the presence of conductive fibers in the matrix. It can be shown that the real part of the impedance R depends on the conductivity of the composite material. *R_m_* and *R* can be considered proportional to the matrix conductivity *σ_m_* and to the conductivity of the composite material *σ*, respectively. They can be used to estimate the concentration of the fibers by means of a simple mixture rule. In order to overcome the drawback of the sensitivity to moisture conditions, the so-called matrix normalized conductivity is used:

(1) $$\frac{R_{m}}{R}=\frac{\sigma }{\sigma_{m}}=1+\left[\sigma_{\text{fibers}}\right]\;V_{f}$$

where *V_f_* is the fiber volume fraction and *σ_fibers_* is the intrinsic conductivity of the fibers which, in the case of highly conductive fibers, only depends on their aspect ratio.

The method has been extensively employed to assess the influence of the fresh state performance on the dispersion of the fibers [6], clearly highlighting the better uniformity in fiber dispersion achievable through FR-SCC. Any direct quantitative comparison between, e.g., the concentration of fibers, as evaluated from Equation (1) and data obtainable from destructive tests (e.g., crushing samples, separating and weighing fibers) could hardly be found in the literature. The need of a dedicated expensive instrumentation, required by the width of the employed frequency range, and the sensitivity of the method to the contact impedance between the electrodes and the structure surface, stand, so far, as the main hindrance to a wide application of the method at the industrial scale.

In order to overcome this problem, the authors have developed a method [17] based on the evaluation of the equivalent capacitance between the two electrodes of a probe which has to be simply laid on the surface of the structure under test. The technique allows one to obtain information about the average fiber concentration and their average direction. The measurement frequencies are relatively low (up to few hundreds of kilohertz) so that the required instrumentation is easily available and relatively inexpensive. However, the method suffers from high sensitivity to humidity and to the coupling between the electrodes and the specimen.

Lataste *et al.* [18] employed a method based on low frequency resistance measurement, with a four electrode arrangement, aimed at reducing the effects of the poor electrical coupling. The method has been demonstrated to be effective in detecting orientation characteristics of the dispersed fiber reinforcement, because of the different resistance measured along the two directions at right angles to each other; a qualitative correlation with mechanical properties measured along to the same directions as above was also provided [8]. The method is by the way not able to provide any quantitative information about the local average concentration of the fibers. This is mainly due to the strong sensitivity of the concrete matrix resistivity to aging, moisture content and presence of electrolytes in the pores which also affects the measured resistivity, beside the effect of fiber concentration.

The effects of conductive fibers on the dielectric properties of the fiber reinforced composite have led to the development of another method, based on the measurement of the effective permittivity through a coaxial probe and microwave reflectometry techniques [19]. The local average fiber concentration could be assessed by assuming a random orientation and because of the known fiber geometry (aspect ratio) which governs their capacitive behavior. However the method is not sensitive to a preferential alignment of the fibers.

Methods based on the study of heat transients and on the effect of fibers on the thermal diffusivity of the composite have been also tentatively applied for the non-destructive assessment of fiber concentration [20]. Temperature fields within a structural element can be easily surveyed through IR thermography, but the slow propagation of temperature variations in thick members, which makes it difficult to provide a controlled input excitation over large areas, may limit the applicability of the method.

Computed Axial Tomography (CAT) scanning allows effective 3D visualization of the fiber network inside a FRC specimen [7,21]. Limits on specimen size together with the need of dedicated equipment and of image analysis software for processing the collected data are so far the main concern in promoting the method for wider use, especially at the industrial scale.

Methods which employ a probe sensitive to the magnetic properties of the steel fibers has been recently proposed and validated [22–24]. They rely on the fact that the steel fiber reinforced concrete is a mixture of two materials with very different magnetic permeability. The presence and the arrangement of the fibers modify the path of the magnetic field lines generated by the winding of a probe which results in a variation of its inductance. Fiber concentration can be quantitatively assessed by calibrating the method. Also the preferential orientation of the fibers can be estimated [11,12]. The method, besides its good sensitivity and robustness, is also characterized by an intriguing ease of use, due to the very simple probe which just needs to be laid of the surface of a structural element. The probe can be easily used even on sub-vertical elements, such as wall and columns, or slabs accessible only from the bottom, without any dedicated care about the coupling. The authors also tested a twin coil probe with the attempt of investigating the dissipative effects due to the alternating magnetic field which invests the steel fibers [25,26].

## 3. Results and Discussion

As mentioned above, Steel Fiber Reinforced Concrete is a composite material consisting of two phases, the concrete matrix and the dispersed fiber reinforcement, each one featuring very different magnetic and electrical properties. While the concrete matrix has a low conductivity and a relative magnetic permeability close to one, the steel fibers are conductive and have a strong ferromagnetic behavior. In general, steel fiber reinforced concrete can be regarded as a sparse mixture, since the volume fraction of the steel fibers is fairly low. Reference will be made, in laying out the theoretical background of the proposed methodology for non-destructive analysis of fiber dispersion in FRC elements, to a representative volume element of the material which is at least three to five times larger than the size of the inclusions (the fibers), which are assumed to be all identical. Under the assumption that the wavelengths involved in the magnetic and electrical phenomena underlying the measuring method, are much larger than both the length of the fibers and the average fiber spacing, this portion of material can be regarded macroscopically homogeneous featuring its own well-defined electrical and magnetic properties, namely magnetic permeability, electric permittivity and conductivity. Since the steel fibers are very long and thin, they can be approximated as ellipsoids, with their polar axis much larger than the equatorial diameter. In this case, under the further assumption that nonlinear effects in the interaction between the matrix and the inclusions can be neglected, closed form expressions of the effective electrical and magnetic properties can be found in literature [27].

As a matter of fact they can be expressed as a function of the properties of the two phases, through a simple mixture rule, in which the contribution of the inclusions depends on the geometrical parameters of the fibers (e.g., fiber length and diameter), on their concentration and finally on their orientation distribution function. It is furthermore important to remark that if the fibers are not randomly distributed, the electromagnetic properties become dyads.

The aforementioned statements anyway lead to the conclusion that it is possible to extract information about the amount and the preferential alignment of the steel fibers from suitable measurements of the electrical and magnetic properties of the composite material, at the scale of a representative volume element.

As highlighted in Section two, a large amount of research work did focus on the characterization of steel fiber reinforced concrete elements by estimating electrical quantities which depend on the effective conductivity or electric permittivity. Much less attention has been paid to magnetic phenomena, even though it is well known that the presence of (ferromagnetic) steel fibers in the concrete matrix may significantly modify the magnetic behavior of the composite, as measured.

**Figure 1.**
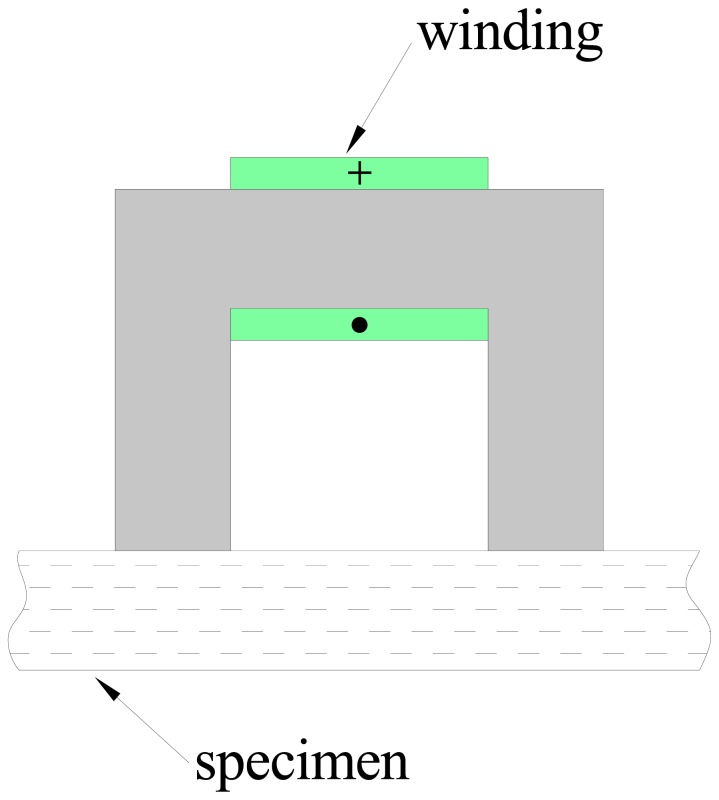
Single winding probe.

**Figure 2.**
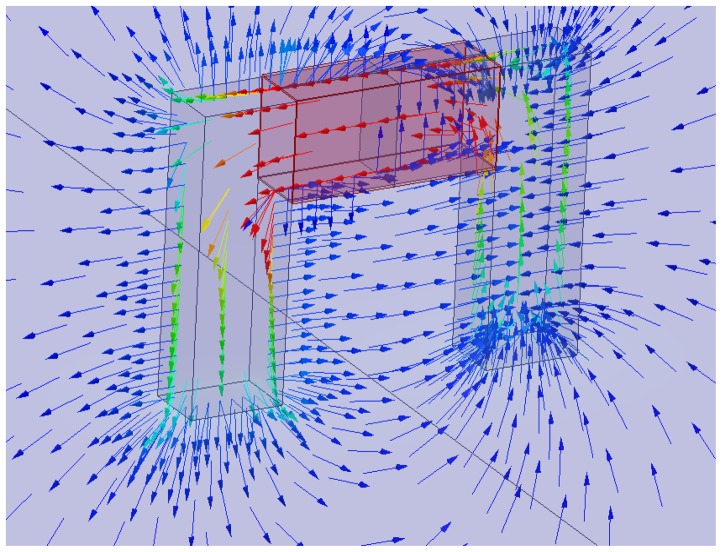
Single winding probe: flux density predicted by FEM analysis.

This has led the authors to investigate the possibility of estimating the fiber concentration and average orientation by measuring a parameter which depends on the effective magnetic permeability of the mixture. To this purpose a low-cost measurement system has been developed and employed. It consists of a probe, a coil wound on a C-shaped ferrite core, as schematically shown in Figure 1, and a customized data acquisition system, which allows the inductance of the winding to be estimated at different frequencies. The flux density predicted by a magnetostatic FEM analysis is shown in Figure 2.

The measurement procedure is extremely simple: the probe has to be laid on the surface of the specimen, and the evaluation of the inductance has to be performed. Its value is clearly affected by both the fiber concentration and orientation in the specimen. The dependence on fiber concentration can be easily understood considering that the presence of steel fibers increases the effective magnetic permeability of the composite material according to a simple mixture rule, which results in an increase of the inductance with respect to that measured when the probe is moved away from ferromagnetic objects. The procedure to assess the effect of the fiber orientation has to take into account that the electromagnetic properties of the composites, as remarked above, becomes dyadic if the fibers are not randomly oriented. In case of a preferential alignment of the fibers, by rotating the probe on the specimen and measuring the inductance along different directions, a dependence of the measured inductance on the probe orientation will be detected: the direction along which the maximum value of the inductance is measured will most likely coincide with the preferential alignment of the fibers. Furthermore, the amplitude of the inductance variation can be employed to evaluate the degree of orientation of the steel fibers.

## 4. Magnetic Method: Experimental Artifacts and Improvement

As extensively discussed in the literature the presence of steel fibers in a cementitious matrix affects the effective magnetic permeability of the composite material. This phenomenon has been exploited to develop the measurement technique for non-destructive analysis of fiber dispersion and orientation in FRC elements as described in the previous section. Basically, a target portion of the specimen is invested by an alternating magnetic field, and the value of the probe inductance depends on the concentration and average orientation of the steel fibers.

It has to be remarked that, when an alternating magnetic field is applied to a ferromagnetic, conductive material such as steel, power losses also occur. It is well known that those losses are related to two phenomena. The first is the magnetic hysteresis of steel, the second is due to the eddy currents which are induced in the steel fibers because of the time-varying magnetic field. As a matter of fact, eddy current losses strictly depend on the geometrical parameters of the conductor exposed to the magnetic field. Under the reliable assumption that the local concentration of the steel fibers is low, well below the percolation threshold, so that the fibers are not touching each other, eddy current losses are just due to the currents induced in each fiber. Since the fibers are small, also the eddy current losses are small. If, on the contrary, the local concentration of the fibers in any part of the specimen is high, namely close to or higher than the percolation threshold, adjacent fibers are likely to be in contact with each other, and this result in a long range connection between the same fibers. The presence of these conduction paths dramatically increases the local power losses.

**Figure 3.**
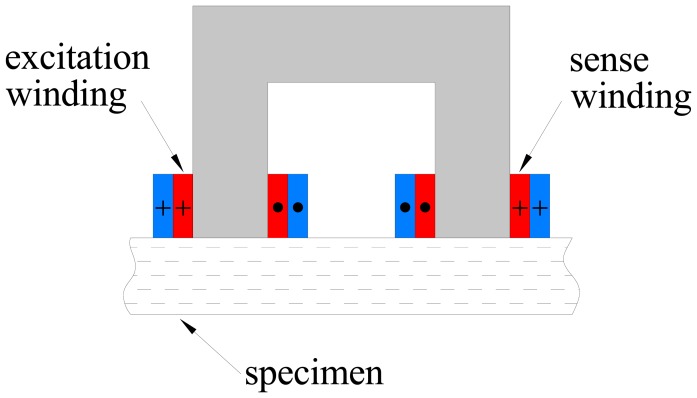
Two winding probe.

**Figure 4.**
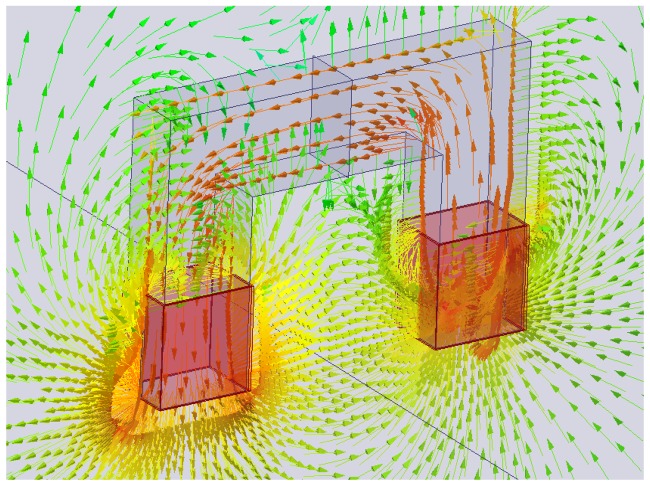
Two winding probe: flux density predicted by FEM analysis.

Because of the aforementioned statements, a simple method to detect the presence of fiber clumps has been implemented using a customized “improved” version of the probe, which will be described hereafter. As a matter of fact, when the probe, as described in the previous sections, is placed on a target section of the FRC element under investigation which is likely to contain fiber clumps, an increase of the electrical power drawn by the winding is expected. However, severe measurement problems arise. The reason is that the losses due to the presence of the steel fibers are very small with respect to those due to the resistance of the winding and to the alternating magnetic field in the ferrite core. In order to overcome these limitations, a new, more sophisticated probe (Figure 3) was developed. It has two windings: the first one generates the magnetic field, while the second is employed to pick up the induced electromotive force. The excitation winding is split into two coils which are wound around each leg of the probe. This arrangement is likely to increase the fraction of the magnetic flux linked with the coils which hits the material under test. The sense winding is placed over a coil of the excitation winding in order to reduce the leakage reactance. Figure 4 reports the flux density predicted by FEM magnetostatic analysis. The measurement of the electromotive force induced in the pickup winding and of the current flowing through the excitation winding allows the losses due to the alternating magnetic field to be directly estimated. However, it is still not possible to separate the power losses due to the steel fibers from those due to the ferrite core. Under the assumption that the flux density in the core would not be affected by the presence of the steel fiber, the power losses due to steel fibers only could be estimated by evaluating the power losses when the probe is laid on the specimen and subtracting those measured when it is placed away from ferromagnetic objects. Unfortunately the hypothesis above is not true, since the ferromagnetic behavior of the steel fibers in the specimen increases the flux density in the core. Therefore, the increase of the power losses due to the alternating magnetic field which is measured when the probe is placed on a SFRC specimen is only partially related directly to the losses occurring in the steel fibers, while the rest is caused by the increase of the core losses. In order to avoid this problem, the sense winding can be employed to set up a proper feedback controller which imposes the voltage across it, hence its flux linkage. In this case the variation of the flux density in the ferrite core due to the steel fibers can be minimized, which furthermore results in a small change of the core losses. Thanks to the two-winding probe with a closed loop set-up, the difference between the losses due to the alternating magnetic field when the probe is placed on the specimen and those evaluated when it is placed away from any ferromagnetic material provides a good estimate of the power losses occurring in the steel fibers alone.

## 5. Experimental Results

**Figure 5.**
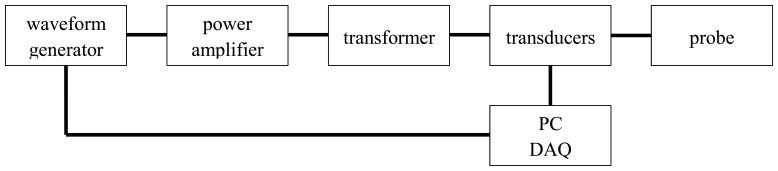
schematic view of the test set-up.

The performance of the single winding magnetic probe has been widely tested by the authors. The excitation system consists of a sine wave generator connected to a power amplifier and a transformer, while the measurement of the voltage and current is provided by a data acquisition board (DAQ) (Figure 5). A personal computer controls both the excitation and the measurement process, while providing the computation of the inductance at different frequencies through a spectral analysis of the voltage and current signals.

**Figure 6.**
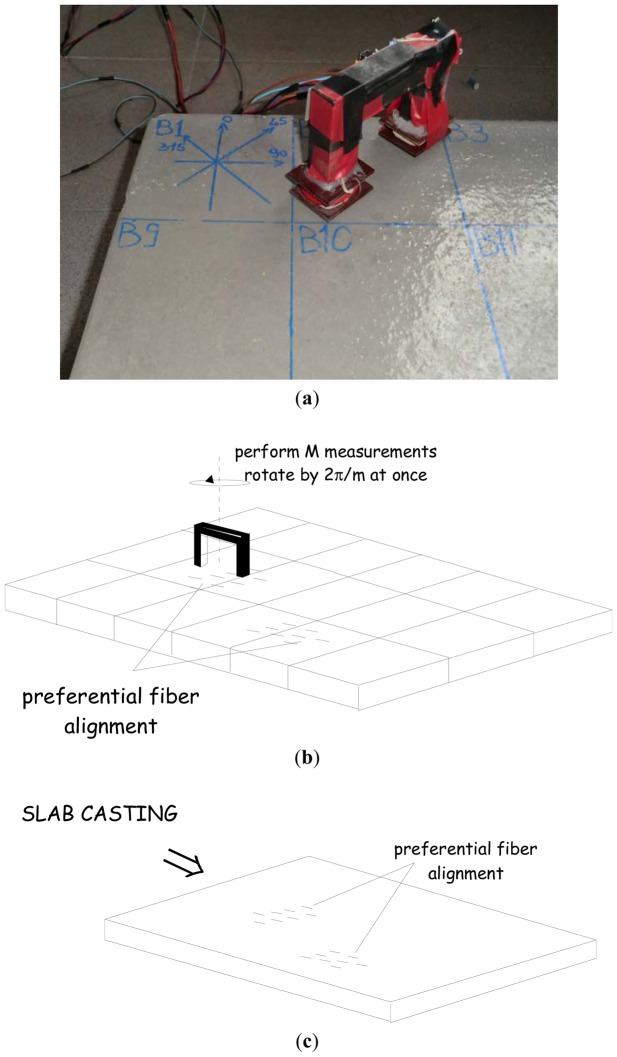

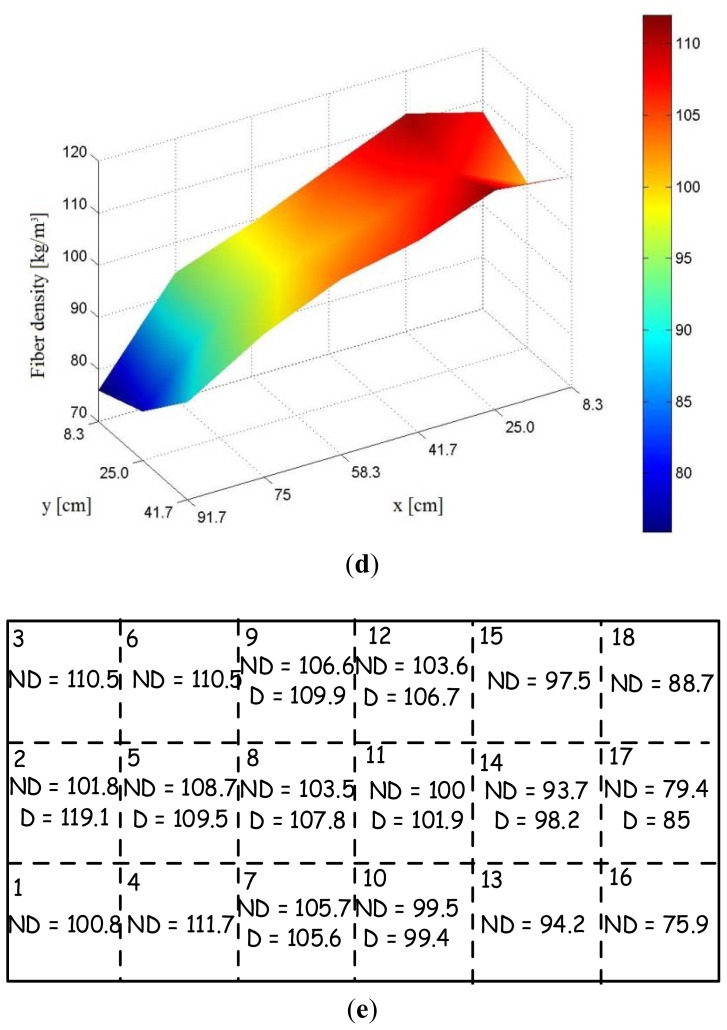
Magnetic measuring sensor for ND characterization of fiber dispersion and orientation in FRC slabs (**a**); schematic of the measuring procedure (**b**); casting of a FRC slab specimen featuring preferential fiber alignment (**c**); ND estimated fiber dispersion cartography (**d**) and comparison between ND estimated and destructively assessed local fiber concentration (**e**).

By employing the set-up described above a thorough characterization of fiber dispersion and orientation of Steel Fiber Reinforced Concrete slabs was performed in a very simple way (see in Figure 6(a) an image of the employed sensor placed on a SFRC slab element). It is worth here remarking that the specimen was cast with a FRCC containing 100 kg/m^3^ steel fibers, 13 mm long and with a diameter equal to 0.16 mm.

First of all, the probe has to be placed away from ferromagnetic or conductive objects and the inductance L0 of the winding has to be evaluated. Then a reference grid is set-out on the slab surface, e.g., dividing it into N equal square sectors (Figure 6(b)), large enough to have representative volume elements and to accommodate the probe. In order to perform the measurements a reference direction has to been chosen. A common choice can be the casting direction, which is likely to be the most probable average orientation of the steel fibers (Figure 6(c)). Each sector has to be analyzed by placing the probe in its center so that the two legs are aligned with the reference direction and then measuring the inductance L. In order to evaluate the dependence of the inductance L on the likely alignment of fibers, the probe can be subsequently rotated by a prescribed angle Δφ (e.g., 1/Mth of 180°), and the inductance measured once again for each different angular position of the probe: the sector will be completely analyzed once a 180° rotation of the probe will have been performed. Once the process has been repeated for each and all the N sectors of the reference grid drawn on the FRC slab surface, a set of M•N inductance values will be obtained. The compensated inductance values [23]*ΔL* can be easily computed as the differences between the inductance values L of the composite and the reference value *L_0_*. It can be shown that *ΔL* contains reliable information about the concentration of the steel fibers and their preferred direction. It is furthermore worth to notice that its value is almost independent on the frequency of the excitation signal in a range between a few hundred hertz and few kilohertz.

For each sector M compensated inductance values are thus available. Previous work [11] has shown that their average value *ΔL_average_* is strictly related to local fiber concentration: for fiber volume fractions commonly employed in the engineering practice, it has been shown that the average compensated inductance value can be assumed to be proportional to the local fiber concentration. This allows a calibration of the aforementioned dependence to be easily performed, which then provides a quantitative tool to perform the non-destructive estimate of the local fiber concentration [11], the results of which can be plotted as in Figure 6(c). The results obtained have proven to be consistent both with those obtained from other non-destructive and destructive techniques (Figure 6(d)), and, as it will be detailed hereafter, with the mechanical properties of the material, measured on specimens featuring a geometry consistent with the reference grid [12].

**Figure 7.**
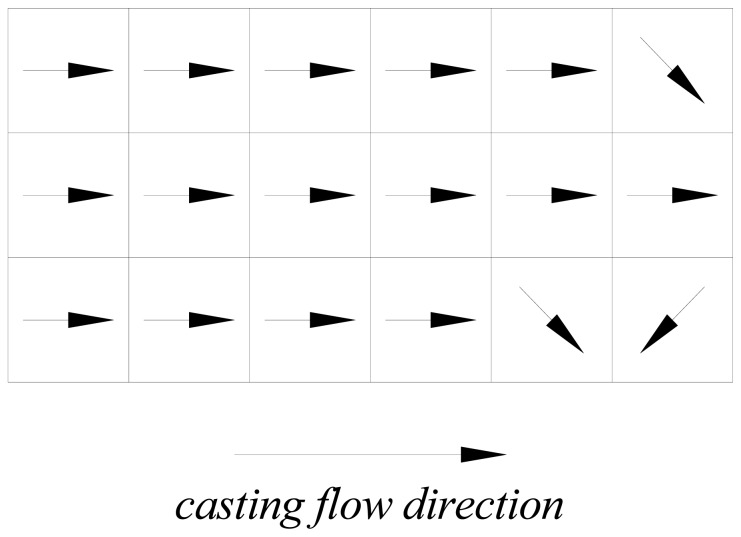
Direction of the maximum inductance in each sector of the slab.

As mentioned above, the method developed by the authors also allows to get information about the preferential orientation of the fibers. Under the aforementioned assumptions underlying the measurement methodology and technique, it is self-evident that the direction along which the maximum value of the compensated inductance is measured is likely to coincide with the preferential alignment of the fibers. For the same slab, cast as shown in Figure 6(a) a simple graphical representation of the direction along which maximum compensated inductance was measured for each sector is shown in Figure 7. A most likely preferential alignment of the fibers consistent with the flow kinematics of the employed casting process appears.

Experimental tests performed on specimens subjected to tensile stress either parallel or orthogonal to the preferential alignment of the fibers confirmed a stronger dependence of the mechanical toughness properties of the fiber reinforced composite material and the orientation of the fibers [12]. This has been estimated either on the fracture surface, at the end of the tests, through image analysis techniques or, as a bulk value in each specimen, through the “fractional compensated inductance”, suitably defined as follows, in analogy with data processing employed by other authors with reference to other non-destructive fiber dispersion analysis techniques:

(2) $$f_{⫽}=\frac{2\Delta L_{⫽}}{\Delta L_{⫽}+\Delta L_{⊥}}$$

where ΔL_//_ and ΔL_⊥_ are the compensated inductances corresponding to the parallel and orthogonal direction with respect to the casting flow. A deeper insight into this issue will be provided in the forthcoming section.

**Figure 8.**
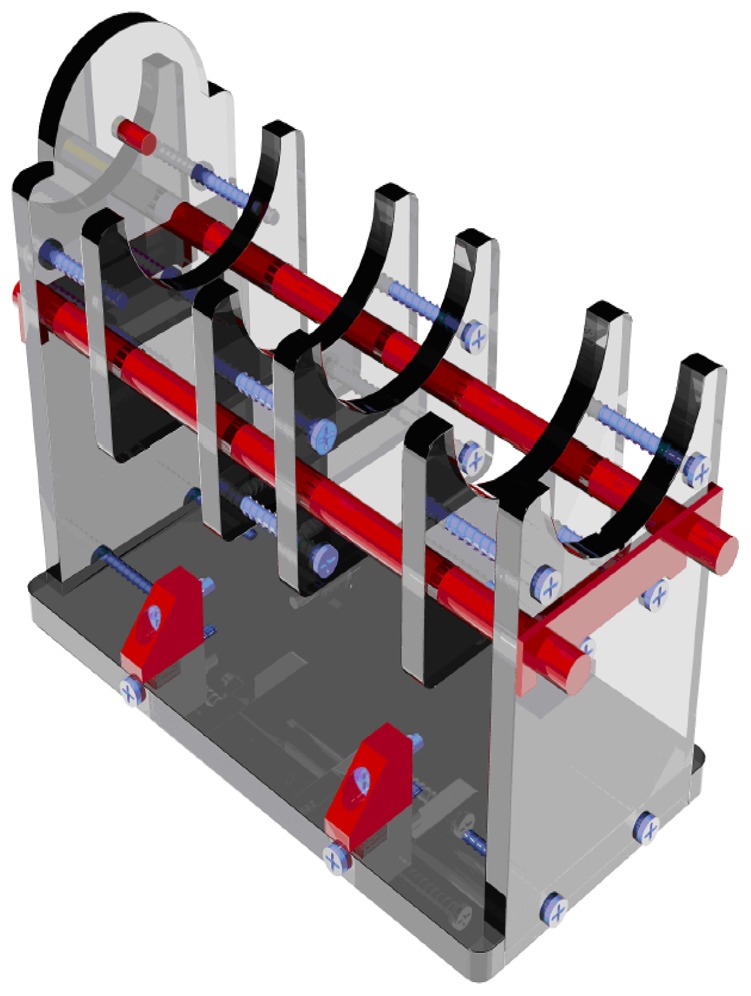
Support allowing the test of cylindrical specimens.

**Figure 9.**
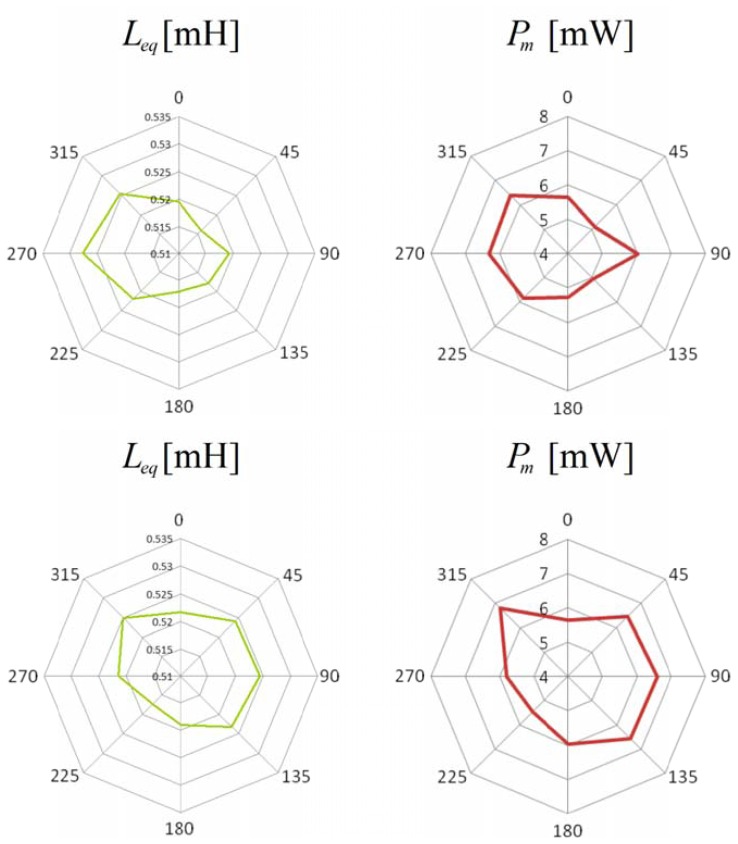
Polar plots of the self-inductance and of the power losses due to the alternating magnetic field measured on two different cylindrical specimens.

The two winding, closed loop probe described in the previous section has been also employed to study the local concentration of the steel fibers in cylindrical specimens obtained by core drilling. Because of the casting process, the steel fibers are assumed to be randomly oriented. The probe has to be placed on a suitable support (Figure 8), which allows to test the cylinders. Once a reference “radial direction” has been traced on one basis of each specimen, the cylinder under test has just to be placed on the support so that the radial mark is aligned with respect to the magnetic axes of the coils. The system automatically performs the measurement of the self-inductance of the excitation winding, of the mutual inductance between the two windings and of the losses due to the alternating magnetic field at different frequencies while maintaining almost constant the amplitude of the flux linked with the sense winding. Once the measurement task is terminated, the specimen has to be rotated by a prescribed angle, as usual a fraction of π, and the measurement has to be run again. The test ends when a full rotation of the cylinder has been completed. The polar plots of the self-inductance Leq, of the mutual inductance Lm and of the power losses Pm are generally provided as the output of the measuring process. It is clear that the self-inductance and the mutual inductance are both heavily affected by the fiber concentration in the material between the legs of the probe. In some cases the plot of the power losses is quite similar to those of the inductances, while, in other cases, it has a different shape (Figure 9). The authors hypothesize that those differences arise when a fiber clump is located between the legs of the probe. In fact, the presence of conductive paths between the steel fibers generates a significant increase of the losses due to eddy current effects. Further research by the authors is ongoing in order to investigate the relationship between the losses and the fiber clumping.

## 6. Correlation to Material Properties: Addressing a “Structure Reliability” Perspective

**Figure 10.**
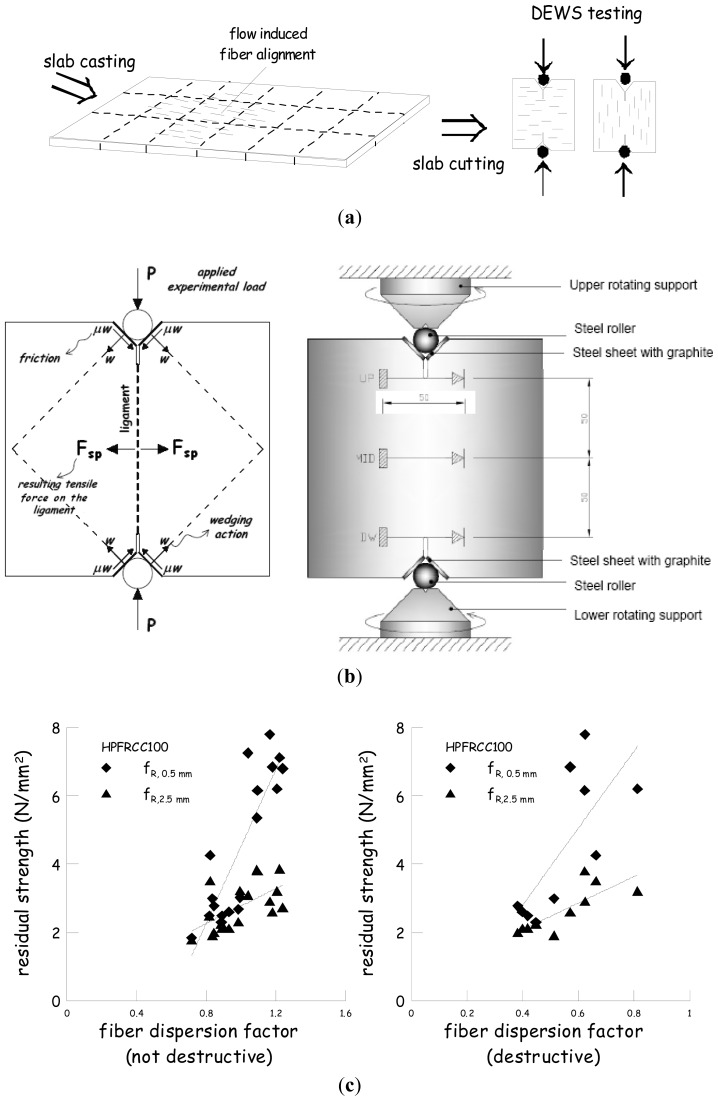
Schematic of slab casting and specimen cutting for Double Edge Wedge Splitting (DEWS) test (**a**); specimen geometry and test set-up (**b**); residual stresses *vs.* fiber orientation/dispersion factor (**c**).

The aforementioned correlation between fiber dispersion/orientation and mechanical performance of fiber reinforced cementitious composites cast a new light on the significance of the analysis of fiber dispersion itself in FRC elements from an engineering application perspective. An example of how this correlation could be established is given hereafter. The same slab shown in Figure 6(a) was cut into square tile specimens, according to the same reference grid drawn on its surface for the measurement process. The specimens were employed to perform the so-called Double Edge Wedge Splitting Test [12,28,29], which, due to the suitable test geometry, allows the material tensile fracture behaviour and toughness to be measured in a direction either parallel or orthogonal to the main fiber alignment (Figure 10(a)). As a property of the material, the residual tensile stresses at prescribed values of the crack opening, measured as sketched in Figure 10(b), can be assumed: Figure 10(c) shows residual stresses at crack openings of 0.5 and 2.5 mm, plotted *vs.* a fiber dispersion orientation factor, defined from the measurements garnered through both non-destructive magnetic survey and a destructive check, by means of image analysis of specimen fracture surface and specimen crushing and fiber separation. As a matter of fact, the local “fiber dispersion factor” was calculated as the product between the local fiber density, either destructively or non-destructively evaluated, dimensionless to the nominal one, and, respectively, either the fiber orientation factor, calculated with the number of fibers on the fracture surface, or the fractional compensated inductance, defined as in Equation (2), along the direction orthogonal to the same fracture surface.

The correlation between fiber dispersion related parameters and mechanical properties of the composite material is evident: once it has been assessed, through a preliminary characterization testing, which has to be performed beforehand for each application, the fiber dispersion/orientation plot, garnered from the ND magnetic survey as shown in Figure 6, could be converted, by means of an educated estimate, into a plot, which shows the spatial dispersion of the material properties, which highlights potentially weaker zones, because of poorer fiber dispersion.

This would also allow to tailor, e.g., the finite element prediction of the mechanical performance of the intended structural application, not limiting it to a mere homogeneous and isotropic description of the material behaviour and not even to a spatial dispersion of material properties featuring a random scatter, according to prebuilt-in options. On the contrary, a dispersion of material properties coherent with the surveyed dispersion of the fibers could be assigned, coupled, in case, with a transversely isotropic constitutive model of the material, which should allow the effects of preferential fiber alignment to be taken into account and which may affect the performance of the structure, whereas biaxial membrane stress states are likely to be experienced, as a function of its geometry, boundary and loading conditions. This will finally allow a thorough and reliable prediction of the structural performance, as affected by the dispersion and, in case, tailored orientation of the fibres.

## 7. Conclusions

In this paper an overview has been provided of the research activity performed by the authors all along the last lustrum in the field of non-destructive analysis of fiber dispersion in Fiber Reinforced Concrete Elements by means of low frequency electrical and magnetic methods.

A method based on inductance measurements has been developed and calibrated and was shown to be effective in detecting local concentration as well as, in case, orientation of the fibers. The technique has been progressively improved, as also described in detail, in order to garner more reliable and robust information about the aforementioned fiber dispersion related issue.

As a matter of fact a simple C-shaped ferrite core with a coil wound on it has been employed as an early version of the probe: when an alternating current flows through the coil, a magnetic field is generated. By placing the probe on the surface of a FRC element, the dispersed fiber reinforcement, as a function of its local average concentration and orientation in the sensed region (between the legs of the C-probe) does affect the magnetic field and hence the values of the measured magnetic properties, namely the self-inductance of the winding, which can thus be employed to estimate the same fiber dispersion and orientation characteristics. An improved version of the probe, featuring a twofold exciting and sensing winding, and a closed loop architecture, has been also implemented. As a matter of fact it allows both the inductance and the power loss to be measured, the last one being possibly correlated to the presence of fiber clumps. Further related work is on-going.

Finally, a correlation of results on the fiber dispersion characteristics and mechanical properties of the fiber reinforced composite has been shown: this cast an interesting light over the significance of the proposed survey and measuring methodology in an engineering application perspective, which has also been addressed in the paper.
